# Periodontal health in a population with Parkinson's disease in Spain: a cross-sectional study

**DOI:** 10.4317/medoral.25540

**Published:** 2022-10-16

**Authors:** Ana María García-de-la-Fuente, Aitziber Fernández-Jiménez, Irene Lafuente-Ibáñez-de-Mendoza, Mari José Lartitegui-Sebastián, Xabier Marichalar-Mendia, José Manuel Aguirre-Urizar

**Affiliations:** 1 Department of Stomatology II. University of the Basque Country (UPV/EHU), Spain; 2 Department of Nursing I. University of the Basque Country (UPV/EHU), Spain

## Abstract

**Background:**

The aim of this research is to evaluate the periodontal health of patients with Parkinson Disease (PD) in a Spanish cohort.

**Material and Methods:**

A cross-sectional study was performed on 104 patients with PD (mean age: 66.19+9.3 years) and 106 controls (mean age: 59.26+14.11 years). A pre-designed clinical protocol was implemented, which included a standardized epidemiological index for periodontal disease (CPITN), clinical attachment loss (CAL), tooth-loss, full mouth plaque index (FMPI), and oral hygienic habits. Univariate descriptions and comparative analysis were performed.

**Results:**

The majority of PD patients presented good oral hygienic habits. There were no significant differences in relation to CPITN, periodontitis, gingival recessions and tooth loss. However, moderate/severe CAL (*p*=0.027) and FMPI (*p*=0.003) was higher in the PD group.

**Conclusions:**

There were no differences on periodontitis and tooth loss between both groups. The higher number of advanced CAL and presence of biofilm in the PD group could be related to the difficulties to perform an effective tooth brushing due to this neurological disorder.

** Key words:**Parkinson’s disease, periodontal disease, clinical attachment loss, oral hygiene.

## Introduction

Parkinson's disease (PD) is one of the most common neurological diseases (1), affecting 6.2 million people worldwide (2). Prevalence of PD increases with age, being the incidence in Spain similar to that in Europe (3).

Periodontitis is considered the sixth most prevalent disease in the word and has a great impact on oral health, including tooth loss and edentulismo (4,5), which negatively affect the quality-of-life patients (6). Biofilm control is a strong factor in the prevention and establishment of periodontitis, reducing the clinical attachment loss (CAL) (7).

Previous reviews have already analysed the oral health of patients with PD, with only a few focusing on periodontitis and with controversial results (8,9). Patients with PD characteristically develop motor and cognitive alterations that affect their manual skills (tremor, rigidity, bradykinesia, postural instability...), which contribute to a poorer plaque control and a greater periodontal disease (10). The periodontal analysis of these studies has been performed with different tools: CPITN index (11,12), CAL (13,14), probing pocket depth (PPD) (14-16), bleeding on probing (BOP) and gingival index (12-14,16), gingival recessions (REC) (14), gingivitis (14,17), tooth mobility (14,16) and plaque index (PI) (13,14,16,18). The risk of PD and periodontitis was associated on a recent cohort study (19), suggesting that control of periodontitis could play an important role in the prevention of PD.

With this background, we designed this study in order to analyse the periodontal health in a cohort of Spanish patients diagnosed with PD.

## Material and Methods

- Study design and patients

This cross-sectional study was performed on 210 patients from the Basque Country (Spain), 104 with PD and 106 without PD. Patients of the PD group (PDG) were members of the Parkinson's Association of Bizkaia (ASPARBI). Individuals in the control group (CG) were recruited among relatives of the test group (n=47) and patients from the Dental Clinic Service of the University of the Basque Country (UPV/EHU) (n=59), until a similar sample size was reached.

All the included patients were 18 years-old. The inclusion criteria for the PDG were having a confirmed diagnosis of Parkinson's disease by a Neurology Service, and for the CG not being diagnosed with Parkinson's disease, or any other neurological disease.

This study was approved by the Research Ethics Committee of the UPV/EHU (CEISH/246/2015), and was conducted in accordance with the international ethics standards of the Declaration of Helsinki of 1975 (revised in 2013). A written informed consent was obtained from all the participants.

- Data compilation

Prior to the study, sessions were held to homogenize the assessment criteria among the different clinical assessors (AMGF, ILIM, MJLS and JMAU), in order to achieve maximum concordance. All patients were evaluated by at least two trained clinical examiners (AMGF, ILIM, MJLS and JMAU). Discrepancies were resolved by consensus and in cases where this was not possible, a third assessor (AMGF, JMAU) was always involved. In all cases, a complete anamnesis and periodontal examination was performed with a plane dental mirror and a standardized periodontal probe (PCP-11, Hu-Friedy, Mfg. Co. LLC, Chicago, USA).

For the extraction of data, a specific protocol was designed that included: demographic data (age, gender), behaviour or social habits (smoking history), oral hygiene habits (toothbrushing frequency, interproximal hygiene with daily use of interdental brushes and/or dental floss, use of mouthwashes and/ water flosser, presence of bleeding with habitual toothbrushing), and perception of patient’s oral health.

The subjective oral hygiene perception was categorized into 3 options: “bad or poor”, “regular” or “good”. Although supragingival control plaque is a key factor to maintain oral health, and surfaces with highest standards of plaque control (10%-20%) should be compatible with oral health, we decided that PI 25% was a more realistic value for general population. Thus, according to the full mouth plaque index (FMPI) (20), objective oral hygiene was divided into three categories: good ( 25%), moderate (25%-50%) and bad (50%).

The periodontal exploration protocol included the following epidemiological index and data:

1) Community Periodontal Index of Treatment Needs (CPITN) (21) at 6/sites/tooth in all teeth of the sextants, with the exception of third molars, recording the highest value of the included teeth in the sextant; in those cases where the patient was edentulous or it was recorded the presence of a full implant-supported prosthesis, the CPITN of this patient was categorized as “not valuable”.

2) Patient’s full mouth plaque index (FMPI) (20).

3) Clinical attachment loss (CAL; distance in mm from the cementoenamel junction to the bottom of the periodontal pocket), which was categorized into 3 groups: mild (1-2 mm), moderate (3-4 mm) or severe (5 mm) (22).

4) Gingival recessions (REC; distance in mm from the cementoenamel junction to the gingival margin) (23), including if these were local or generalized.

5) Dichotomous index for the presence of peri-implant disease (24).

6) Variable “Tooth-Loss” (TL) referred to the total number of natural teeth lost.

7) Periodontal disease was categorized as: a) “none” or “health”: absence of CAL or CPITN=0 and clinical inflammation (CPITN=1), b) “gingival disease”: presence of inflammation without CAL (CPITN=1), c) “periodontitis”: presence of ≥2 interproximal sites with CAL 2 non-adjacent teeth. Third molars were excluded.

- Statistical analysis

The statistical analysis was performed with IBM SPSS v.23 software by a blinded statistical examiner. A univariate description was performed, describing the quantitative (mean and standard deviation) and qualitative variables (frequency and percentage). For the analysis, Pearson Chi-square test was used when the two variables were qualitative or categorical; the Fisher exact test was used when the expected frequencies were too low. The Student's t-test when one variable was quantitative and the other qualitative. It was considered statistically significant when *p*<0.05.

## Results

- Sociodemographic characteristics and behaviour or social habits.

A total of 104 patients diagnosed with PD (PDG), corresponding to 66 men (63.5%) and 38 women (26.5%) with mean age of 66.2+9.3 years, and 106 individuals without PD (CG) corresponding to 37 men (34.9%) and 69 women (65.1%) with a mean age of 59.2 + 14.1 years, were analysed. In the CG, 36 women were relatives of the PDG patients. Differences in age and gender between the study groups were significant (*p*<0.001).

The number of smokers was significantly lower in patients with PD (*p*<0.001), but alcohol consumption was similar. Tooth loss was the biggest oral problem stated by patients with PD (17.3%), followed by periodontitis (12.5%) (Table 1).

The majority of patients in both groups reported brushing their teeth more than twice a day, with the average daily brushing being lower in the PD group. Interdental hygiene was also lower in PDG (*p*<0.023). Interdental brush was the most common tool used in both groups. Respectively, 3.8% and 1.9% of PDG patients brushed their teeth with an electric toothbrush and/or an oral irrigator (Table 2).

When analysing the oral hygiene according to age, no differences were observed between the groups, except for the patients under 65 in the CG, who flossed more frequently. In the intra-group analysis of PDG, 60.5% of women brushed their teeth more than twice a day (*p*=0.016) and 59.9% used interdental hygiene devices (*p*<0.000), both dental floss (*p*<0.009) and interdental brush (*p*<0.02) (Table 2). Nearly 50% of patients in both groups used mouthwashes, with alcoholic mouthwashes being more frequent than non-alcoholic (*p*<0.021) (Table 2).

Most of the participants in both groups subjectively considered their oral hygiene to be good or fair. Regarding gender, 56.1% of men in the PDG considered their hygiene”fair", compared to men in the CG group, whose perception was that they had "good hygiene" (48.6%) (*p*=0.015). Within each group, a higher percentage of women rated their oral hygiene as "good" (*p*<0.010) (Table 2). None of the participants reported the need of extra help or the assistance of caregivers to clean their mouth.

- CPITN index, Periodontal disease, FMPI and tooth loss (TL).

CPITN index and number of patients with periodontitis, peri-implant disease and REC were higher in the PDG, without significant differences (Table 3). Patients over 65 years had greater periodontal disease, which was significant in the PDG group (*p*<0.007) (Table 3).

CAL was higher in the PDG group in comparison to healthy controls, with 84% of them showing "moderate-advanced" CAL (3 mm) (*p*=0.027) (Table 3). No differences on age and gender were observed between PD and control patients when analysing the severity of CAL. However, patients older than 65 years had a higher PPD in both PDG (*p*<0.02) and CG (*p*<0.046).

The mean FMPI was higher in the PDG (*p*=0.003), with differences amongst men and women (*p*=0.033). In both groups the mean FMPI value was lower in females, being significantly higher in males of the PDG group (*p*=0.041) (Table 3).

The FMPI showed a "good" (FMPI<25%) objective oral hygiene in 12.3% of the CG and 2.9% of the PDG patients, with a higher percentage of "poor" hygiene (FMPI>50%) in both groups (*p*=0.004). When analysing these data according to gender, women showed better results (PDG 65.8% vs CG 58%) than men (PDG: 83.3% vs CG: 64.9%) in both study groups (*p*=0.011). (Table 2)

There was slightly higher mean TL in patients with PD, and in those over 65 years of both the PDG (*p*<0.001) and the CG (*p*=0.03). Similarly, the mean number of sextants showing TL was higher in patients in the PDG, especially in the posterior area (Table 4).

**Table 1:**
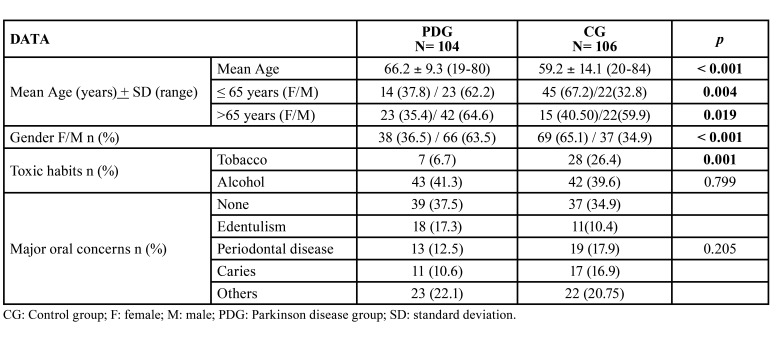
Total data of the study groups.

**Table 2:**
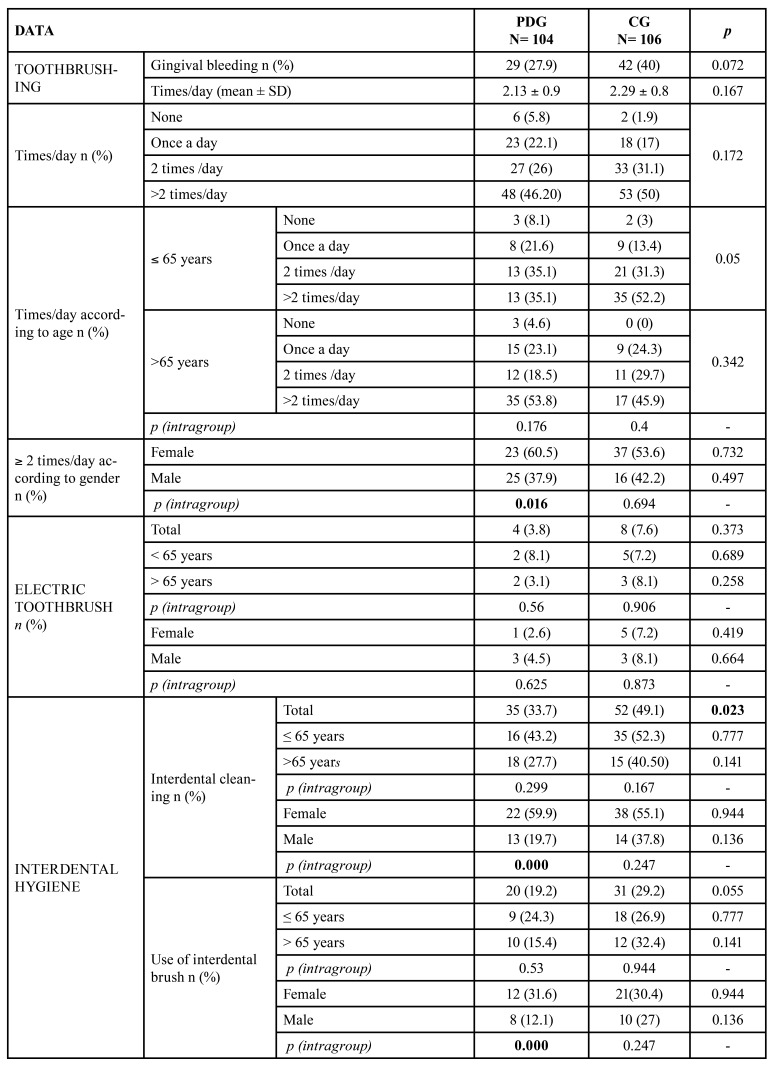
Data of oral hygiene habits and subjective hygiene of the study groups.

**Table 2 cont.**
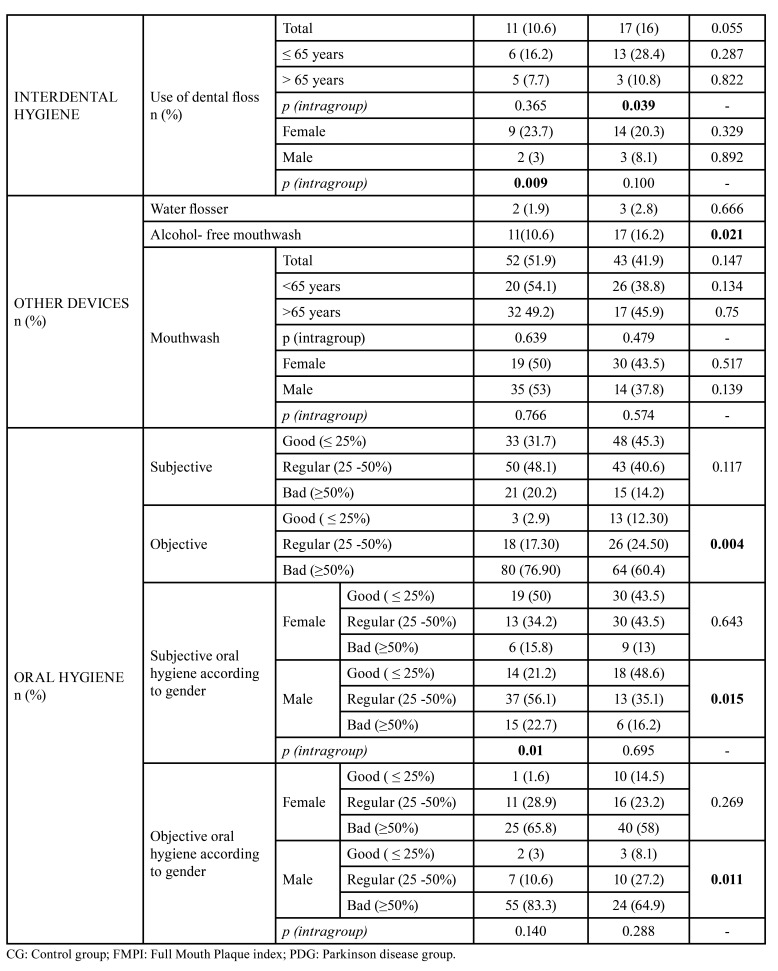
Data of oral hygiene habits and subjective hygiene of the study groups.

**Table 3:**
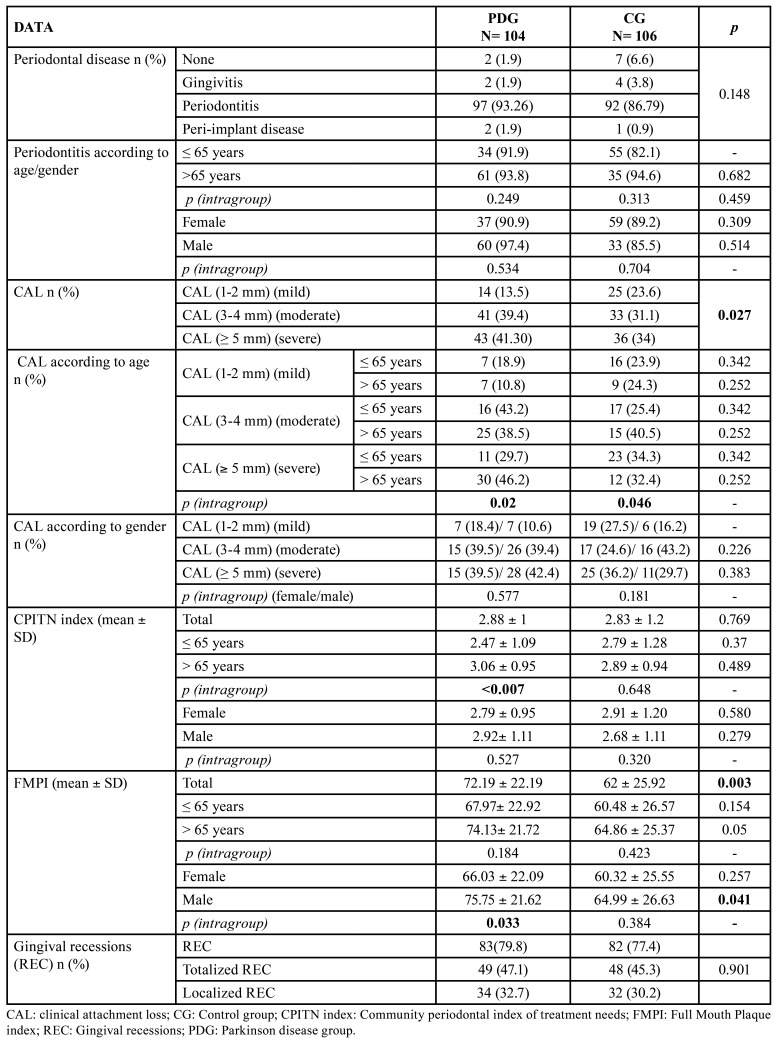
Data of periodontal pathology, CAL, CPITN, FMPI of the study groups.

**Table 4:**
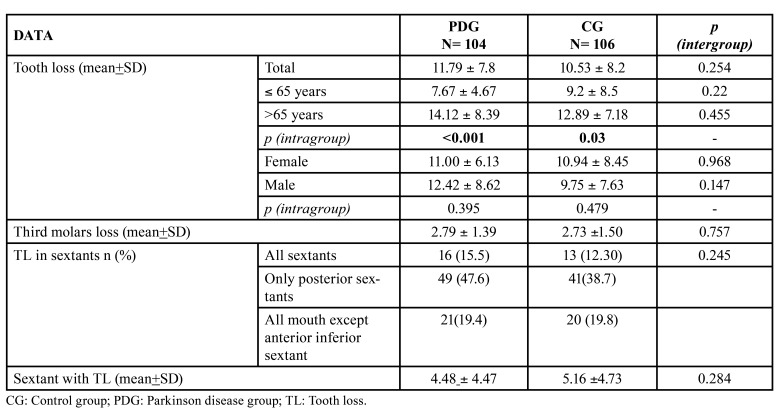
Data of tooth loss of the study groups.

## Discussion

Parkinson's disease is a neurodegenerative pathology whose prevalence has increased in recent years2. In comparison to individuals without PD, few studies (9,10,25) have described a greater periodontal and oral pathology in these patients, but with limited samples and controversial results. Due to the heterogeneity of previous findings, we considered comparing our results only with the studies in which a clinical oral examination was performed (11-18).

Our research reaffirms that PD affects mostly men, with a similar ratio of 2:1 to that observed by Pradeep *et al*. (13), who are older than 60 years as in previous studies (11,13,14,17). The CG had a majority of women (n=69), who mostly belonged to the inner familiar circle of PD patients (18) and the Dental Clinic Service of the EHU, which is one of the top demanding Dental Care Service in Spain.

Presence of periodontitis was high in both groups and slightly higher in the PDG (93.26% vs 86.79%), as well as the CPITN index (*p*=0.148). These results don’t match previous studies (11,13-16,18), where the number of patients with PD and periodontitis doubled the CG. We consider that these differences are linked to different methodologies used on the studies and the time when they were performed.

When analysing the CPITN, we found significant differences between both groups in relation to age, which was higher in the PDG (*p*=0.007). We didn’t observe such discrepancy according to gender, contrary to Schwarz *et al*. (11), where women had a higher risk of suffering from periodontal disease. CAL was statistically more common in PDG patients (*p*=0.027). At the intra-group analysis, CAL was also higher in patients over 65 years in both groups, same as previous studies (11,13-16,18).

The increase of periodontal pathology amongst advanced-age PD patients is linked to a higher plaque index; although 72.2% of them reported brushing their teeth more than twice a day. This accumulation of biofilm is linked to the neurological alterations derived from the progression of the disease and motor impairment (13,16). Previous studies (13,14,18) also observed high levels of plaque despite good oral hygiene and use of dental floss or mouthwashes (13,18). Achieving a correct removal of interdental plaque to minimise the risk of periodontitis and dental caries requires a great skill, which justifies, as in the general population (26), our patients' preference for interdental brushing over flossing.

Although women in the PDG reported good oral hygiene (interdental brushes and/or floss), we did not recognise a significant lower CAL. It has been recognised that 29-57% of patients with PD have difficulties with self-care (10,13,18,27), in addition to increasing age. No PDG patient reported this handicap, contrary to previous studies where 3-35% weren’t able to perform independent oral hygiene (10,18).

This finding might be associated to the lack of dexterity due to progression of PD and hypokinesia, but not to the characteristic "tremor" (13). These motor difficulties to manipulate objects have been well recognised in Spanish patients with PD (56%). High levels of biofilm could be the first sign of neurological and motor impairment in individuals with good oral hygiene (13). Thus, it is very important for dentists to acknowledge the incapacity of adult patients to perform proper oral hygiene, as this may be a diagnostic warning sign of a neurodegenerative disease such as PD, or its aggravation (Pradeep *et al*., 2015).

These circumstances would explain why, although mean age, brushing and interdental hygiene were similar in our study groups, PDG patients had significantly worse plaque control and a bigger number of severe periodontitis. This ineffective plaque removal in PDG patients, which seems to be linked to the syndrome's own alterations, justifies the recommendation of the use of electric toothbrushes and the correct periodontal maintenance, in order to preserve a good periodontal and oral health. The dental professional has a key role in the motivation, diagnosis and treatment of patients with PD, whose prevalence is expected to keep increasing in the future, by individualising the dental care of each patient depending on the general condition of the disease.

Finally, the number of missing teeth was similar in both groups but higher in the PDG. This was lower than those obtained previously (14), which we consider reflects the concern for the oral health care and maintenance of the population.

The main limitation of our study is that, due to the advanced age of many PDG patients and the limitation imposed by the disease itself, we didn’t perform a correct standardised periodontal examination (X-ray study and periodontal chart). It was therefore decided to make a clinical diagnosis (basic periodontal examination) of periodontitis based on the CPITN (21) and CAL, according to the 1999 Armitage classification (22). Presence of CAL in at least 2 non-adjacent teeth, together with general or localised periodontitis, was taken into account (22). Another limitation was the time of data collection in the GC, which was severely affected by the SARS-COV II pandemic.

The highlight of our study is the large sample of patients that we analysed, both with PD and CG, which allowed us to compare the different oral alterations. On a clinical practice level, the implications of our research recommend the development of personalised guidelines for PDG patients, their families and caregivers. These patients require a good dental care with regular diagnostic assessment, and a correct oral and supportive periodontal therapy.

In summary, patients with PD don’t have a bigger prevalence of periodontitis or TL than those without PD. However, they do have a higher CAL, probably related to the inefficient biofilm control (psychomotor skill) caused by the progressive neurological alterations of the disease. In an adult patient with good oral hygiene habits, difficulty to perform an efficient plaque removal could be a warning sign for PD. Longitudinal cohort studies with long follow-up periods would be necessary to corroborate these findings.
